# Supplementary material for: Testis-specific serine kinase 6 (TSSK6) is abnormally expressed in colorectal cancer and promotes oncogenic behaviors
Source: J Biol Chem. 2024 May 16;300(6):107380. doi: 10.1016/j.jbc.2024.107380 (PMC11214309; doi:10.1016/j.jbc.2024.107380)
Supplement: Supplemental Figures S1–S3 Legends [file mmc1.docx]

Supplemental Figure Legend:

Figure S1: A. Graph of TSSK6 mRNA expression across human tissues derived from the GTex database. B. Oncoprint of TSSK6 mRNA-expressing tumors (pink) with tumor type indicated based on TCGA PanCancer Atlas Study. C. Patient outcome data for indicated tumors based on TSSK6 mRNA median expression. D. TCGA CRC cases exhibiting the top and bottom quartile of TSSK6 mRNA expression and associated mutation of indicated genes. P-value calculated by chi-squared analysis (Prism Graphpad) Software.

Figure S2: A. Zoomed cross section of human testes tissue stained with anti-TSSK6. Red labelling indicates TSSK6-positive cells and black labelling indicates TSSK6-negative cells. Scale bar represents 100 μm. B. Indicated HCEC lysates resolved by SDS-PAGE (10%) and immunoblotted with anti-TSSK6. C. Mouse spermatogonial cell lysates were resolved by SDS-PAGE (14%) and membranes incubated +/- immunizing peptide. D. Full blot image of indicated lysates resolved on 14% SDS-PAGE and probed with an anti-TSSK6 antibody. Arrow indicates non-specific band at ~100kDa.

Figure S3: DLD-1 xenograft tumors +/- V5-TSSK6 (Figure 5B) were harvested, fixed and stained using the TSSK6 IHC protocol. Scale bar represents 100 μm.
