# Supplementary material for: Testis-specific serine kinase 6 (TSSK6) is abnormally expressed in colorectal cancer and promotes oncogenic behaviors
Source: J Biol Chem. 2024 May 16;300(6):107380. doi: 10.1016/j.jbc.2024.107380 (PMC11214309; doi:10.1016/j.jbc.2024.107380)

Delgado et al
Supplemental Figure 1

A.

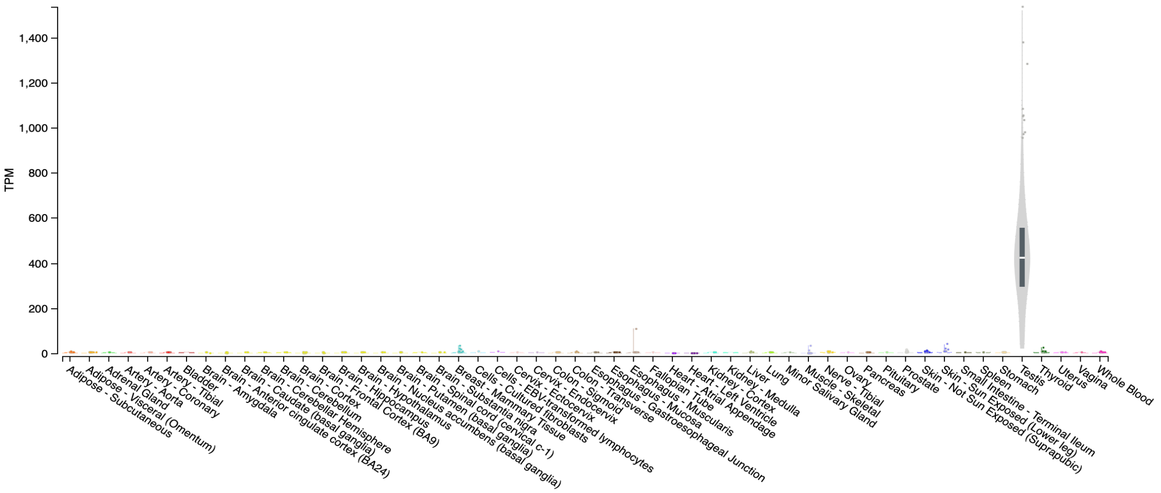

B.

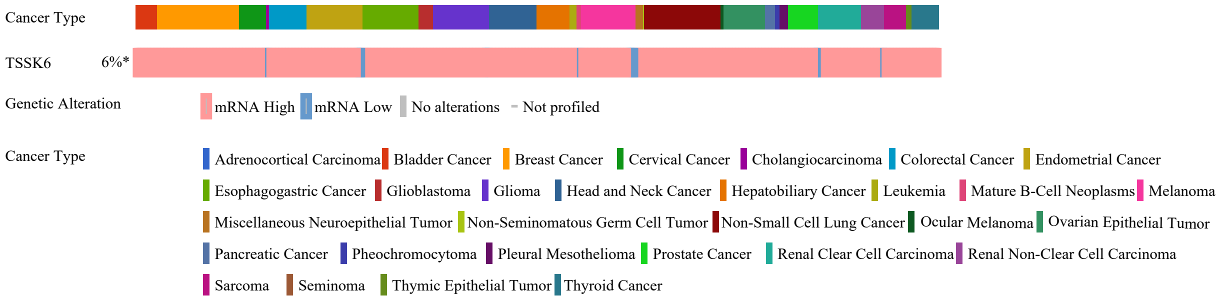

C.

Tumor Type	Survival	# Patients	Hazard Ratio	p-Value	Survival	# Patients	Hazard Ratio	p-Value
Breast	RFS	2032	0.99	0.92	OS	943	1.13	0.38
Ovarian	RFS	614	0.95	0.5609	OS	655	0.99	0.56
Lung	FP	874	1.22	0.074	OS	1411	0.79	0.003
Gastric	FP	522	1.06	0.63	OS	631	1.22	0.074
Pancreatic	DFS	278	1.1	0.435	OS	1189	0.9	0.128
**Colorectal**	**RFS**	**1130**	**1.35**	**0.0092**	OS	809	1.11	0.39

RFS: Relapse Free Survival
DFS: Disease Free Survival
FP: Time to First Progression
OS: Overall Survival

D.

KRAS			
TSSK6 population:	Mutant # cases	Wild-type # cases	chi-square p-value
Top quartile	18	33	0.0727
Bottom quartile	27	24	
**APC**			
TSSK6 population:	Mutant # cases	Wild-type # cases	chi-square p-value
Top quartile	33	18	0.8368
Bottom quartile	32	19	
**p53**			
TSSK6 population:	Mutant # cases	Wild-type # cases	chi-square p-value
Top quartile	29	22	0.2347
Bottom quartile	23	28	

Delgado et al
Supplemental Figure 2

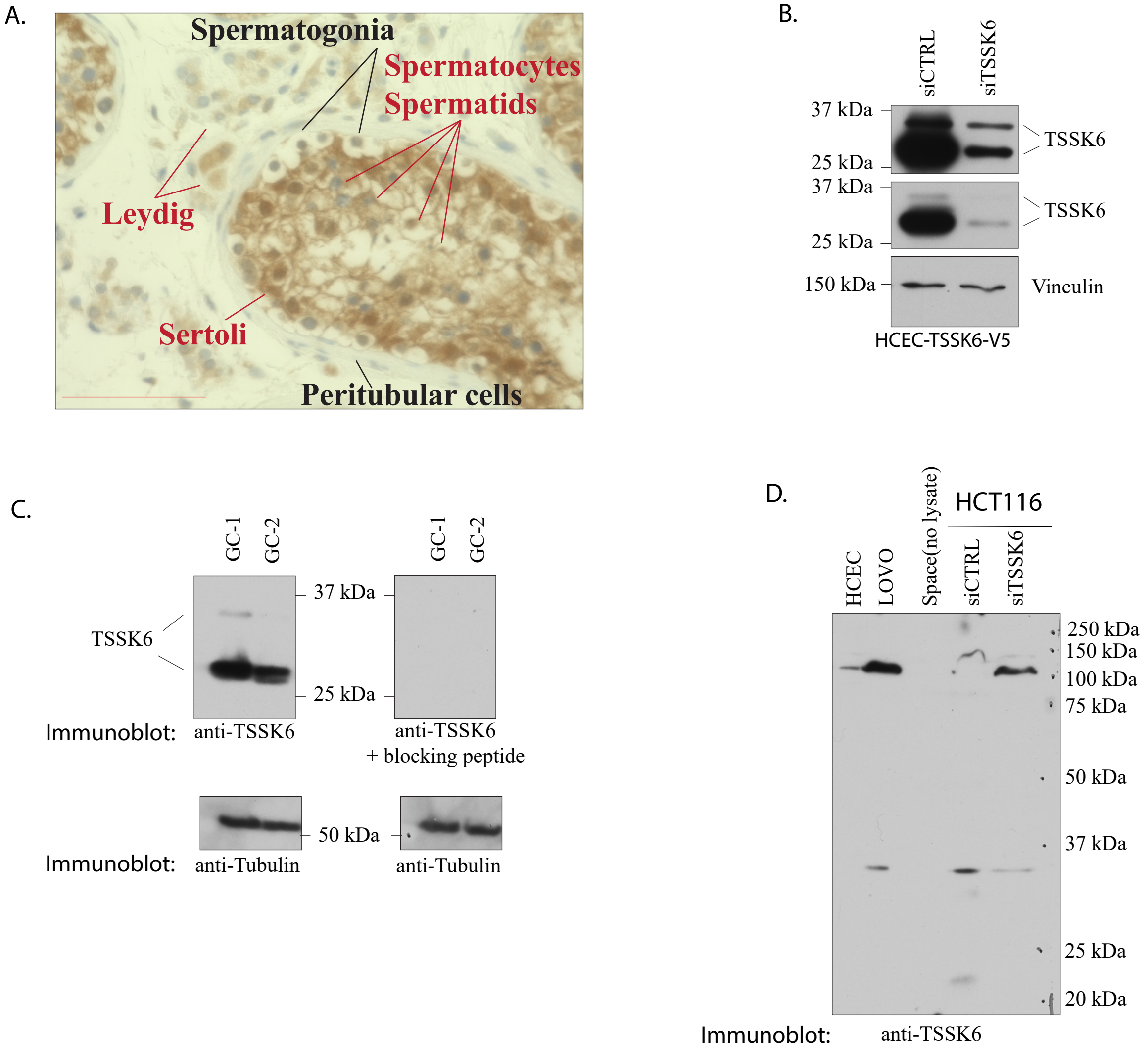

Delgado et al
Supplemental Figure 3

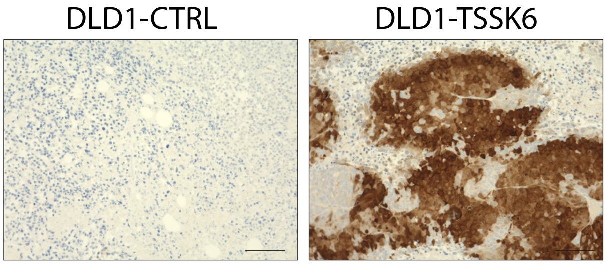

Supplement: Supplemental Figures S1–S3 [file mmc2.pdf]
